# Comprehensive analysis of the cerebrospinal fluid and serum metabolome in neurological diseases

**DOI:** 10.1186/s12974-024-03218-0

**Published:** 2024-09-26

**Authors:** Carolin Otto, Rea Kalantzis, Dorothee Kübler-Weller, Andrea A. Kühn, Tina Böld, Armin Regler, Selina Strathmeyer, Johannes Wittmann, Klemens Ruprecht, Steffen Heelemann

**Affiliations:** 1 Department of Neurology, Charité – Universitätsmedizin Berlin, corporate member of Freie Universität Berlin and Humboldt-Universität Zu Berlin, Charitéplatz 1, 10117 Berlin, Germany; 2 Berlin Institute of Health at Charité, Charité – Universitätsmedizin Berlin, Berlin, Germany; 3 Lifespin GmbH, Am BioPark 13, 93050 Regensburg, Germany

**Keywords:** NMR spectroscopy, Cerebrospinal Fluid, Serum, Metabolome, Metabolomics, Neurology, Parkinson’s Disease, Neurodegenerative diseases, Cerebral Ischemia, Multiple Sclerosis, Biomarker

## Abstract

**Background:**

Comprehensive characterization of the metabolome in cerebrospinal fluid (CSF) and serum by Nuclear Magnetic Resonance (NMR) spectroscopy may identify biomarkers and contribute to the understanding of the pathophysiology of neurological diseases.

**Methods:**

Metabolites were determined by NMR spectroscopy in stored CSF/serum samples of 20 patients with Parkinson’s disease, 25 patients with other neuro-degenerative diseases, 22 patients with cerebral ischemia, 48 patients with multiple sclerosis, and 58 control patients with normal CSF findings. The data set was analysed using descriptive and multivariate statistics, as well as machine learning models.

**Results:**

CSF glucose and lactic acid measured by NMR spectroscopy and routine clinical chemistry showed a strong correlation between both methods (glucose, R^2^ = 0.87, n = 173; lactic acid, R^2^ = 0.74, n = 173). NMR spectroscopy detected a total of 99 metabolites; 51 in both, CSF and serum, 16 in CSF only, and 32 in serum only. CSF concentrations of some metabolites increased with age and/or decreasing blood–brain-barrier function. Metabolite detection rates were overall similar among the different disease groups. However, in two-group comparisons, absolute metabolite levels in CSF and serum discriminated between multiple sclerosis and neurodegenerative diseases (area under the curve (AUC) = 0.96), multiple sclerosis and Parkinson’s disease (AUC = 0.89), and Parkinson’s disease and control patients (AUC = 0.91), as demonstrated by random forest statistical models. Orthogonal partial least square discriminant analysis using absolute metabolite levels in CSF and serum furthermore permitted separation of Parkinson’s disease and neurodegenerative diseases. CSF propionic acid levels were about fourfold lower in Parkinson’s disease as compared to neurodegenerative diseases.

**Conclusions:**

These findings outline the landscape of the CSF and serum metabolome in different categories of neurological diseases and identify age and blood–brain-barrier function as relevant co-factors for CSF levels of certain metabolites. Metabolome profiles as determined by NMR spectroscopy may potentially aid in differentiating groups of patients with different neurological diseases, including clinically meaningful differentiations, such as Parkinson’s disease from other neurodegenerative diseases.

**Supplementary Information:**

The online version contains supplementary material available at 10.1186/s12974-024-03218-0.

## Background

There is a need for reliable biomarkers for diagnosis, monitoring of disease activity, and response to therapy in neurological diseases. One approach for the identification of such markers is the detailed characterization of the cerebrospinal fluid (CSF) and serum metabolome by 1H-nuclear magnetic resonance (NMR) spectroscopy (Molina et al., 1997; [3–5, 13, 17]). Indeed, previous metabolomic studies revealed disease specific alterations of metabolites in different neurological diseases. For example, alterations in glutamate and glutamine metabolism were linked to multiple sclerosis (MS), amyotrophic lateral sclerosis, and epilepsy [4]. Branched-chain amino acids (BCAAs), such as leucine, isoleucine, and valine were shown to be elevated in PD and may also play a role in other neurodegenerative diseases [4]. Nevertheless, results of former metabolomics studies were sometimes heterogeneous and an overarching analysis and comprehensive characterization of the metabolome in neurological diseases was hitherto missing.

Here, we systematically analysed, by NMR spectroscopy, the CSF and serum metabolome of patients with degenerative (Parkinson’s disease, other neurodegenerative diseases), inflammatory (multiple sclerosis), and ischemic (cerebral ischemia) neurological diseases as compared to control patients with normal CSF findings. We also studied the influence of age and blood–brain-barrier function on metabolite levels in CSF. Furthermore, we investigated whether and how CSF and serum metabolite profiles may permit to differentiate between different categories of neurological diseases.

## Patients and methods

### Standard protocol approvals, registrations and patient consent

CSF and serum samples were obtained during routine medical work-up and subsequently stored at −80 ° in the Charité CSF/Serum Biobank at the Central Biobank of Charité—Universitätsmedizin Berlin (ZeBanC, Charité Campus Virchow Klinikum, Augustenburger Platz 1, 13353 Berlin, Germany). All lumbar punctures were performed for diagnostic purposes only and with written informed consent of the patients or their guardians. Moreover, written informed consent for storage of samples in the Charité CSF/Serum Biobank and for usage of the stored samples for research purposes was obtained from all study participants. The storage of CSF and serum samples in the Charité CSF/Serum Biobank and usage of stored samples for the present retrospective, observational, single-center study were approved by the institutional review board, Charité—Universitätsmedizin Berlin, Berlin, Germany (EA4/018/17, EA4/008/20).

### Patients

Patients included in this study were identified by searching the database of the Charité CSF/Serum Biobank for patients with diagnoses of Parkinson’s disease (PD), other degenerative diseases (DD), cerebral ischemia (CI), and multiple sclerosis (MS). Furthermore, we included a group of control patients (CP), who had various neurological diseases, but no signs of inflammation in the CSF. Diagnoses deposited in the database were extracted from the patients’ discharge letters and had been made by experienced neurologists at the Department of Neurology, Charité—Universitätsmedizin Berlin, based on clinical, laboratory and imaging findings, applying appropriate current diagnostic criteria. Any additional clinical data, for instance, clinical scores, were obtained from the patients’ medical records. Patients included in this study had to be ≥ 18 years of age. All CSF and serum samples analysed in this study were collected between January 2018 and August 2020.

### Parkinson’s disease

We included 20 patients with PD, 9 of whom had an akinetic-rigid type, 4 a tremor-dominant type and 7 a mixed type. The median (range) Hoehn and Yahr score, a global score for the severity of Parkinson’s disease ranging from 0 (no symptoms) to 5 (wheelchair-bound), was 2 (1–4). The motor scale of the Unified Parkinson's Disease Rating Scale (MDS-UPDRS III), an assessment tool for Parkinson’s disease symptoms ranging from 0 (no symptoms) to 132 (worst), was obtained from all patients. The median (range) MDS-UPDRS III while patients were on their individual dopaminergic medication was 30 (9–52) and the median (range) in the off-state without medication was 55 (37–102). At the time of lumbar puncture, all patients with Parkinson’s disease were treated with dopaminergic treatments (calculated according to [20]) with a median (range) levodopa equivalent dose of 1108 (765–1451) mg.

### Degenerative diseases

The DD group included a total of 25 patients, comprising patients with corticobasal degeneration/progressive supranuclear palsy overlap (n = 3), multi system atrophy (n = 1), unclassified atypical Parkinson syndrome (n = 1), frontotemporal dementia (n = 4), cerebellar ataxia (n = 1), structural epilepsy within global atrophy of the brain (n = 1), primary progressive aphasia (n = 1), gait disorder and cognitive impairment (n = 3), encephalopathy with cognitive impairment (n = 1), cognitive impairment (n = 1), Alzheimer’s dementia (n = 5), and mild cognitive impairment (n = 3).

### Cerebral ischemia

The CI group included 22 patients, who had either brainstem (n = 5) or median cerebral artery ischemia (n = 17). Stroke severity was assessed by the National Institutes of Health Stroke Scale (NIHSS), ranging from 0 (no symptoms) to 42 (severe stroke) and the modified Ranking Scale (mRS), a scale to assess the degree of disability or dependence in daily activities, ranging from 0 (no symptoms) to 6 (dead). The median (range) NIHSS at hospital admission was 1 (0–5) and the median (range) mRS was 0 (0–4). The median (range) interval from onset of symptoms to lumbar puncture was 5 (1–217) days.

### Multiple sclerosis

We included 48 patients with a diagnosis of MS according to the McDonald 2017 criteria [19]. 38 patients had relapsing remitting multiple sclerosis (RRMS) and 10 patients had primary progressive multiple sclerosis (PPMS). The median (range) expanded disability status scale (EDSS) score, a tool to assess disability in patients with MS, ranging from 0 (normal neurological exam) to 10 (death due to MS), was 2.25 (0–8). At the time of lumbar puncture, 18 of the 48 patients received treatment for MS, including interferon beta-1a (n = 1), mycophenolate mofetil (n = 1), intrathecal steroids (n = 5), rituximab and intrathecal steroids (n = 8), and ocrelizumab and intrathecal steroids (n = 3). One patient was previously treated with interferon-beta and natalizumab but was without MS-specific immunotherapy for seven months at the time of lumbar puncture. The remaining 29 patients did not receive any MS-specific immunotherapies at the time of lumbar puncture. Of the 16 patients with intrathecal steroids, 2 had received intrathecal steroids within 2 to 3 days before collection of the CSF/serum samples used for this study. The other 14 patients had received intrathecal corticosteroids ≥ 3 months before CSF/serum withdrawal.

### Control patients

As a control group, we included patients with various neurological diseases, who underwent lumbar punctures for diagnostic purposes, but had no inflammatory CSF findings, as defined by a CSF cell count  ≤ 5/µl and absence of CSF-specific oligoclonal bands. The control group included patients with meningioma of the optic nerve (n = 1), encephalopathy (n = 3), neuropathy/chronic inflammatory demyelinating neuropathy (n = 9), motor neuron disease (n = 1), frontal lobe tumor (n = 1), normal pressure hydrocephalus (n = 1), chorea (n = 1), dystonia (n = 1), acute polymorphic psychosis (n = 1), spinal muscular atrophy type III (n = 1), chronic fatigue (n = 1), narcolepsy (n = 1), idiopathic intracranial hypertension (n = 1), cerebral amyloid angiopathy (n = 1), NMDA-receptor-encephalitis (n = 1), myelitis, encephalitis, encephalomyelitis (n = 2), non-organic sleep disorder (n = 1), neuroborreliosis fully recovered (n = 1), neuromuscular disorders (n = 2), somatoform disorders (n = 15), depression (n = 1), headache (n = 6), migraine (n = 1) and numbness of the skin (n = 4).

### Measurement of routine CSF and serum parameters

All routine CSF parameters were measured at Labor Berlin GmbH, Berlin, Germany. CSF cell count was determined as previously described [23]. CSF glucose was determined photometrically on a Roche/Hitachi cobas c analyser. CSF lactic acid was determined colorimetrically on a Roche/Hitachi cobas c analyser. CSF protein was determined by turbidimetry on a Roche/Hitachi cobas c analyser. Albumin concentrations in CSF and serum were determined by nephelometry (BN ProSpec, Siemens Healthcare GmbH, Erlangen, Germany).

### ^1^H-NMR measurement

Frozen CSF and serum samples were shipped on dry ice to lifespin GmbH (Am BioPark 13, 93,053 Regensburg, Germany, https://lifespin.health/), where they were stored at −80 °C until NMR analyses.

NMR spectroscopy is an analytical method based on the interaction of NMR-active nuclei with magnetic fields. It allows for both the identification and absolute quantification of molecules in a sample. Based on their chemical structure, these molecules give rise to characteristic peak patterns in an NMR spectrum that allows for their identification. Quantification can be achieved by integration of these peaks and comparing them to the integral of a known internal standard.

Measurements were carried out with the lifespin^®^ metabolomic profiling platform using ^1^H-protons. Further use cases, such as animal models, are described elsewhere [2, 22].

For NMR spectroscopy, 230 µL CSF or serum samples were diluted in 300 µl of an aqueous buffer solution, containing p.A. quality H_2_O, 0.1 g/l NaN_3_, 0.067 mol/l Na_2_HPO_4_, 0.033 mol/l NaH_2_PO_4_ (pH-value: 7.15 ± 0.05) and 5% D_2_O as field locking substance. For 5 serum and 5 CSF samples whose volumes were < 230 µl, dilutions were adjusted to 200 µl CSF/Serum and 300 µl buffer, 190 µl CSF/Serum and 340 µl buffer, 150 µl CSF/Serum and 380 µl buffer, respectively. As internal standard, 4 mM trimethylsilylpropanoic acid (TSP) was added to CSF and 6 mM pyrazine to serum samples. From the final solution, 500 μl were transferred to 5 mm Bruker NMR tubes and closed with barcode-caps. The samples were stored at 4 °C until subsequent NMR acquisition, which took place within 24 h of sample preparation.

NMR spectra were acquired on a 600 MHz Bruker Avance III HD/NEO NMR spectrometer equipped with a 5 mm BBI probe. 1D NMR spectra were recorded using a NOESY-presaturation pulse sequence (noesygpprpr1d) with a spectral width of 30 ppm and 98,304 data points. The number of scans was set to 128 and 16, relaxation delays to 6 s and 10 s and temperatures to 298 K and 310 K for CSF and serum samples, respectively.

The obtained spectra of CSF and serum samples were Fourier-transformed with TopSpin software version 4.0 (Bruker Biospin, Billerica, USA). All ^1^H-NMR spectra were automatically phased, and baseline corrected. Proprietary lifespin® profiler software (version 1.4) for CSF and blood biomarker analysis was used for automated detection and quantification of CSF and serum metabolites. All serum metabolites were quantified in mmol/l, except for spectral shape parameter fatty acids and spectral shape parameter lipids, which are given in arbitrary units. All CSF metabolites were quantified in µmol/l.

For each metabolite analyzed, the lower limit of detection (LLOD) was determined based on the metabolite’s concentration in human serum using data available from a large database (lifespin GmbH), containing NMR spectra of > 1000 human serum samples. Furthermore, using lifespin’s proprietary automated profiler test software, NMR spectra (> 10,000) were artificially generated for human serum and additionally used for determination of the LLOD. The thus generated lowest value for a given metabolite in serum was taken as the lower limit of detection. The lower limit of detection of metabolites in CSF was similarly determined, but exclusively based on measurements of metabolites in CSF, i.e. no artificially generated spectra were used for determination of LLODs in CSF. In case metabolite values were below the LLOD, they were set to zero and included into the analyses.

### Statistical analysis

Statistical analysis was performed with R [16]. Descriptive statistics are presented as mean, median, absolute range (min–max), and absolute and relative frequencies. Statistical significance of the data presented in Table 1 was assessed by Kruskal–Wallis Test and Chi-Square Test. Statistical significance of the data presented in Table 2 was determined using Wilcoxon-Mann–Whitney tests. The resulting p-values were corrected for multiple testing (FDR correction) and the corrected p-values translated into * notation as follows: p ≤ 0.001 (***), p ≤ 0.01 (**), and p ≤ 0.05 (*). For effect sizes, fold change and Cohen’s d were determined. For two groups, the fold change was calculated as the quotient of the mean values of both groups, where fold change 1 implies that the mean values are the same, fold change 0.5 indicates that the mean value of group 2 is twice as large as the mean value of group 1. Cohen’s d was calculated by dividing the difference of the means of the respective groups by their pooled standard deviation and taking the absolute value where effects are interpreted as small with a Cohen’s d ≤ 0.5, moderate with a Cohen’s d of 0.5–0.8, and as large for a Cohen’s d ≥ 0.8.

**Table 1:** Demographical findings and results of routine CSF examinations in the different groups of patients

	Parkinson’s disease	Degenerative diseases	Cerebral ischemia	Multiple sclerosis	Control patients	p-value^a^
Number	20	25	22	48	58	–
Age (years)	62 (44–85)	71 (49–82)	55 (35–84)	38 (21–77)	42 (20–82)	< 0.0001
Female/male (% female)	13/7 (65%)	10/15 (40%)	8/14 (36%)	31/17 (65%)	31/27 (53%)	0.1
CSF cell count (cells/µl)	2 (0–6)	2 (0–10)	2 (1–5)	6 (0–85)	2 (0–5)	< 0.0001
CSF lactic acid (mg/dl)	14.3 (10.8–23.9)	16.5 (11.8–21.5)	14.4 (12–21.5)	14.7 (11.2–27)	14.2 (10.6–26.1)	0.1
CSF glucose (mg/dl)	64.5 (52–120)	67 (55–136)	64 (49–166)	63 (53–103)	63 (54–125)	0.4
CSF protein (mg/l)	408.9 (181–788.8)	414 (161.3–1361)	340.6 (174.2–757.8)	372.8 (146–2762)	337.6 (180.3–1237)	0.3
CSF albumin (mg/l)	248 (90–387)	291 (128–1110)	231 (102–480)	222 (83–762)	216 (77–767)	0.5
Q_alb_	5.4 (1.8–9)	6.6 (2.9–25.5)	5.5 (2–10.8)	4.95 (1.6–15.2)	4.85 (1.6–20)	0.2

Data are presented as either absolute numbers or median (range)

^a^Statistical significance was assessed by Kruskal–Wallis tests or Chi-Square test

*CSF* cerebrospinal fluid, *Q*_*alb*_ CSF/serum albumin quotient

For the multivariate analysis, a partial least squares discriminant analysis (PLS-DA) and an orthogonal partial least squares discriminant analysis (OPLS-DA) were performed. A PLS-DA is a supervised classification method that uses class information to detect variables that create maximum separation between these classes. Hereby, a set of components is determined, which contain for each succeeding component the highest possible covariance between the data set X and its labelling Y (e.g., the measured data points and a group classification). The model quality is assessed by the cross-validation parameters R2 (variance explained) and Q2 (predictive capability). R2X and R2Y represent the respective variance explained by the X and Y matrix, and Q2Y indicates the predictive accuracy, with an effective model being indicated by a value of the cumulative parameters R2X, R2Y and Q2Y close to 1. An OPLS-DA is a variant of the PLS-DA where variation in the data matrix that is uncorrelated (i.e., orthogonal) to the categorial vector is split off. The scores plots can be interpreted in the same way as for the PLS-DA, but the y-axis in the scores plot now represents orthogonal variation.

Moreover, as machine-learning algorithm for group separation, Random Forest, which is based on decision-tree theory, was applied and cross-validation was used. Performance of models was evaluated by calculating the area under the curve (AUC) value. For any model building (PLS-DA, OPLS-DA, Random Forest) and for calculating the respective statistical significance, the nearZeroVar function in R was used to remove zero variance predictors, i.e., metabolites that have very few unique values relative to the number of samples.

The correlation between the concentration of metabolites in CSF and serum and age was assessed by Spearman rank correlations using GraphPadPrism version 9.0 for Windows (GraphPad Software, San Diego, California USA).CSF/serum quotient diagrams were likewise created and analysed using GraphPadPrism. When analysing any parameter in CSF, an important question is whether a given parameter has reached the CSF by passive diffusion from the blood. In case of such a passive diffusion, a decrease of the blood–brain barrier function leads to increased concentrations of that parameter in CSF. Blood–brain barrier function is typically assessed by the CSF/serum albumin quotient (Q_alb_), with increasing Q_alb_ values indicating increasing impairment of the blood–brain barrier. By correlating the CSF/serum quotient of a given parameter with the CSF/serum albumin quotient (Q_alb_) in so called CSF/serum quotient diagrams, the association of the CSF concentration of that parameter with blood–brain barrier function can be assessed. An increase of the CSF/serum quotient of a given parameter with increasing Q_alb_ values suggests that the parameter has reached the CSF by passive diffusion from blood. We used Q_alb_ values from routine laboratory measurements for creation of CSF/serum quotient diagrams. The association of the CSF/serum quotient of a given metabolite with Q_alb_ was assessed by Spearman rank correlations.

## Results

### Patients

To characterize the CSF/serum metabolome of different categories of neurological diseases, we analyzed, by NMR spectroscopy, paired CSF/serum samples of 173 patients, including 20 patients with Parkinson’s disease (PD), 25 patients with other degenerative diseases (DD), 22 patients with cerebral ischemia (CI), 48 patients with multiple sclerosis (MS), and 58 control patients (CP) with non-inflammatory CSF findings. Table 1 summarizes the demographics of the 5 different groups of patients. While the median age was higher in PD, DD, and CI than in MS and CP, the female/male ratio did not differ between groups.

Consistent with the known mildly elevated CSF cell count in patients with MS [15], results of routine CSF examinations, including CSF cell count, lactic acid, glucose, protein, albumin and the CSF/serum albumin quotient (Q_alb_), showed a higher median CSF cell count in patients with MS. All other routine CSF parameters did not significantly differ between groups (Table 1).

### Correlation of CSF metabolites measured by the lifespin^®^ metabolomic platform and by routine clinical chemistry

**Fig. 1:**
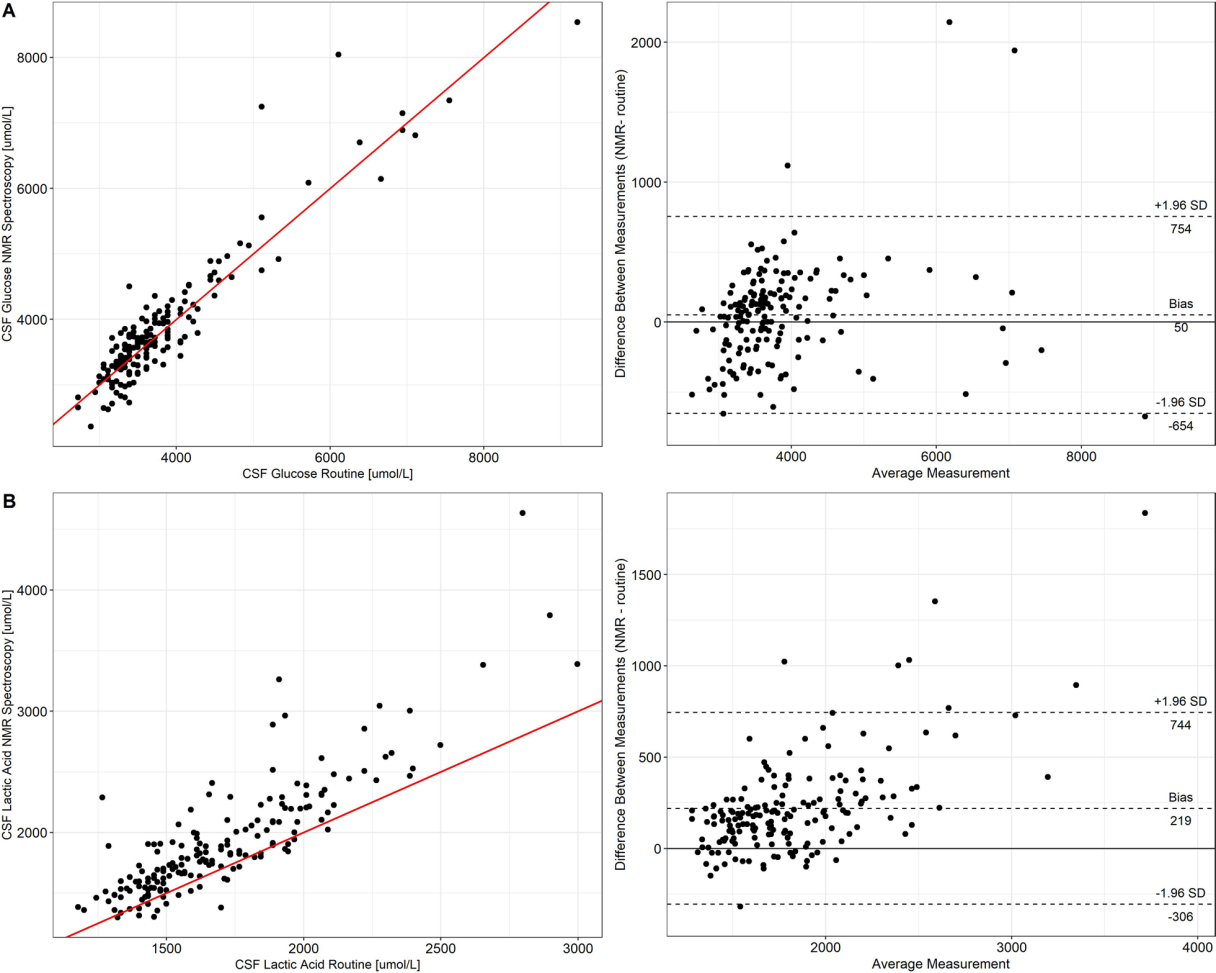
Comparison of CSF glucose and lactic acid measurements by the lifespin^®^ metabolomic platform and routine clinical chemistry. CSF glucose (**A**) and lactic acid (**B**) values obtained by the lifespin^®^ NMR spectroscopy metabolomic platform and routine clinical chemistry were analysed by correlation (left) and Bland–Altman (right) plots. In the correlation plots, the red line shows the least square fit of linear regression. In the Bland–Altman plots, the horizontal axis shows the mean value of the measurements obtained by the two different methods and the vertical axis shows the difference between NMR measurement and routine measurement. Black dashed lines indicate the mean of the differences (also referred to as bias), and the 95% confidence intervals (limits of agreements). The solid black line indicates zero. The actual values are indicated below each line

We first analysed whether the lifespin^®^ NMR spectroscopy metabolomic platform is able to quantify reliably metabolites in CSF. To this end, we compared results of CSF glucose and CSF lactic acid measurements by the lifespin^®^ metabolomic platform with those of routine clinical chemistry measurements. As shown in Fig. 1, results obtained by both methods were strongly correlated (R^2^ = 0.87 for glucose, R^2^ = 0.74 for lactic acid). Likewise, Bland–Altman Plots demonstrated a good agreement between both methods with the average discrepancy between both methods (bias) being relatively small. Additionally, most samples were within the 95% limits of agreement, defined by the mean difference ± 1.96 times the standard deviation. For lactic acid, the difference between the methods tended to get larger with higher values, indicating less method agreement for higher values. Altogether, these findings demonstrate the suitability of the lifespin^®^ metabolomic platform for quantification of metabolites in CSF.

### Analysis of the CSF and serum metabolome by 1H-NMR

The proprietary lifespin^®^ profiler software (version 1.4) for analysis of metabolites in CSF and serum was able to automatically quantify 99 metabolites. Of these, 67 were detectable in CSF, 83 in serum and 51 in both, CSF and serum samples. Accordingly, 16 metabolites were detectable in CSF samples only, and 32 in serum samples only (supplemental Figure S1). Among the 32 metabolites quantified in serum samples only, 16 metabolites were spectral shape parameter lipids, which capture the NMR detectable cholesterol in lipoproteins. A list of the metabolites detected in both CSF and serum, in serum only, or in CSF only is provided in supplemental Table S1.

### Potential confounders

Notably, detection of ethanol and isopropanol in serum and CSF might be due to ethanol and isopropanol containing disinfection agents used during blood or CSF withdrawal. Furthermore, detection of acetic acid might result from the presence of acetic acid in the buffer solution. We also observed correlations between the various spectral shape parameter lipids, which can influence model building (i.e., similar information is given several times as input, hence more variable importance would be placed on this information). Thus, only the spectral shape parameter lipids #1, #7, #12, and #16, which correlate to high density lipoprotein (HDL), low density lipoprotein (LDL), intermediate density lipoprotein (IDL), and very low density lipoprotein (VLDL), were included in the statistical analyses.

For these reasons, we excluded ethanol, isopropanol, and acetic acid in serum and CSF, and all spectral shape parameter lipids except for #1, #7, #12, and #16 in serum from all further analyses. Thus, a total of 84 metabolites, 68 of which were detectable in serum, 64 of which were detectable in CSF, and 48 of which were detectable in both, serum and CSF, were included in the subsequent evaluations.

The median (minimum—maximum) storage time, i.e. the interval between CSF/serum withdrawal and NMR spectrospcopy analysis, of the CSF/serum samples from the 173 patients analyzed in this study was 10 (0—34) months. Analyses by Spearman rank correlations did overall not suggest a relevant influence of storage times up to 34 months on serum and CSF concentrations of the 84 metabolites investigated in this work (Supplemental Table S2).

### Association of CSF/serum metabolites levels with age

We next assessed the association of all 84 metabolites detected in CSF, serum or both with age in the 58 control patients by Spearman rank correlations. For this analysis, all metabolite concentrations were transformed to µmol/l. Altogether, there were 34 significant associations of metabolites in CSF or serum with age (supplementalTable S3, supplemental Figure S2). While CSF or serum concentrations of 31 metabolites increased with increasing age, serum concentrations of only 3 metabolites (citric acid, threonine, albumin) decreased with increasing age. Interestingly, of the 31 metabolites, which increased with age, 22 were present in CSF, while only 9 were present in serum. As the function of the blood–brain barrier decreases with age, these findings may suggest that the increase of certain metabolites in CSF with increasing age might be related to decreasing blood brain barrier function and subsequent increased diffusion of those metabolites from the systemic circulation into the CSF. Thus, we went on to analyze the association of CSF metabolite concentrations with blood brain barrier function.

### Association of CSF/serum metabolites with blood brain barrier function

We created CSF/serum quotient diagrams for all 48 metabolites, which were detected in both CSF and serum, using data of the 58 control patients. Altogether, 18 of 48 CSF/serum metabolite quotients showed significant positive associations with Q_alb_ (supplemental Table S4, supplemental Figure S3), suggesting that those metabolites may reach the CSF at least in part by passive diffusion across the blood brain barrier. Of note, CSF metabolite concentrations of 11 of the 18 CSF/serum metabolite quotients with positive associations with Q_alb_ also increased with age, suggesting that the association of CSF concentrations of these metabolites with age might be due to decreased blood brain barrier function with increasing age.

### Detection rates of metabolites in CSF and serum in the different patient groups

We next analysed the detection rates of the different metabolites among the 5 groups of patients. Detailed lists of the metabolite detection rates (= the number of patients in a given group in whom a given metabolite could be detected/total number of patients in that group) in the 5 different patient groups are provided in supplemental Table S5 (metabolites detected in serum and CSF), supplemental Table S6 (metabolites detected in serum only), and supplemental Table S7 (metabolites detected in CSF only). Interestingly, among the metabolites detectable in CSF and serum, the detection rates in CSF and serum differed quite considerably in some instances. For example, 2-hydroxybutyric acid and ascorbic acid were detectable in CSF samples of all patients, whereas they were less frequently detected in serum. Conversely, proline and serine were detected in serum samples of all patients but were only infrequently detected in CSF. Of note, the metabolite detection rates in serum as well as CSF were overall similar among the 5 different groups of patients (supplemental Tables S5-7).

**Fig. 2:**
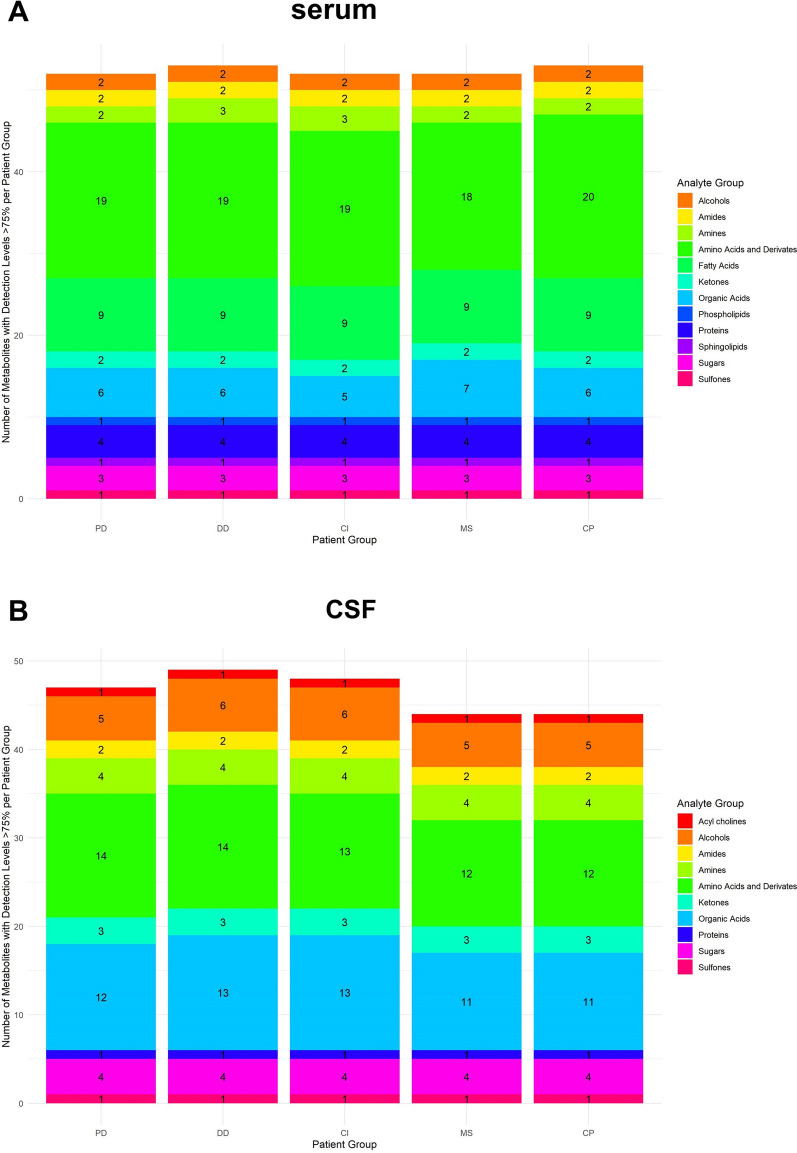
Number of metabolites in serum or CSF detected in > 75% of patients among the different patient groups.The number of metabolites detected in > 75% of (**A**) serum and (**B**) CSF samples is shown for each of the 5 patient groups. Metabolites were grouped into analyte groups as indicated by the color code. *CSF* cerebrospinal fluid, *PD* Parkinson’s disease, *DD* degenerative diseases, *CI* cerebral ischemia, *MS* multiple sclerosis, *CP* control patients

As metabolites that might be specifically associated with a given diseases are expected to be detectable in the majority of patients with that disease, we next analysed how many metabolites were detectable in more than 75% of serum (Fig. 2A) or CSF (Fig. 2B) samples among the 5 groups of patients. Furthermore, to identify possible associations of diseases with specific analyte groups, we investigated the number of metabolites detectable in more than 75% samples by analyte groups. In serum as well as CSF, the numbers of metabolites with detection rates > 75% were overall similar among the 5 patient groups. Furthermore, in serum and CSF, detection rates > 75% among the different analyte groups did not substantially differ among the 5 patient groups. However, the distribution of the metabolites with detection rates > 75% somewhat differed among the different analyte groups between serum and CSF. While the number of organic acids detected with a frequency > 75% was higher in CSF than serum, the number of amino acids and derivatives detected with a frequency > 75% was higher in serum than CSF.

Altogether, in serum as well as CSF, metabolite detection rates among the 5 different groups of patients were overall similar, suggesting that metabolite detection rates may hardly permit to differentiate between the analysed patient groups.

### Multivariate analysis and model building

**Fig. 3:**
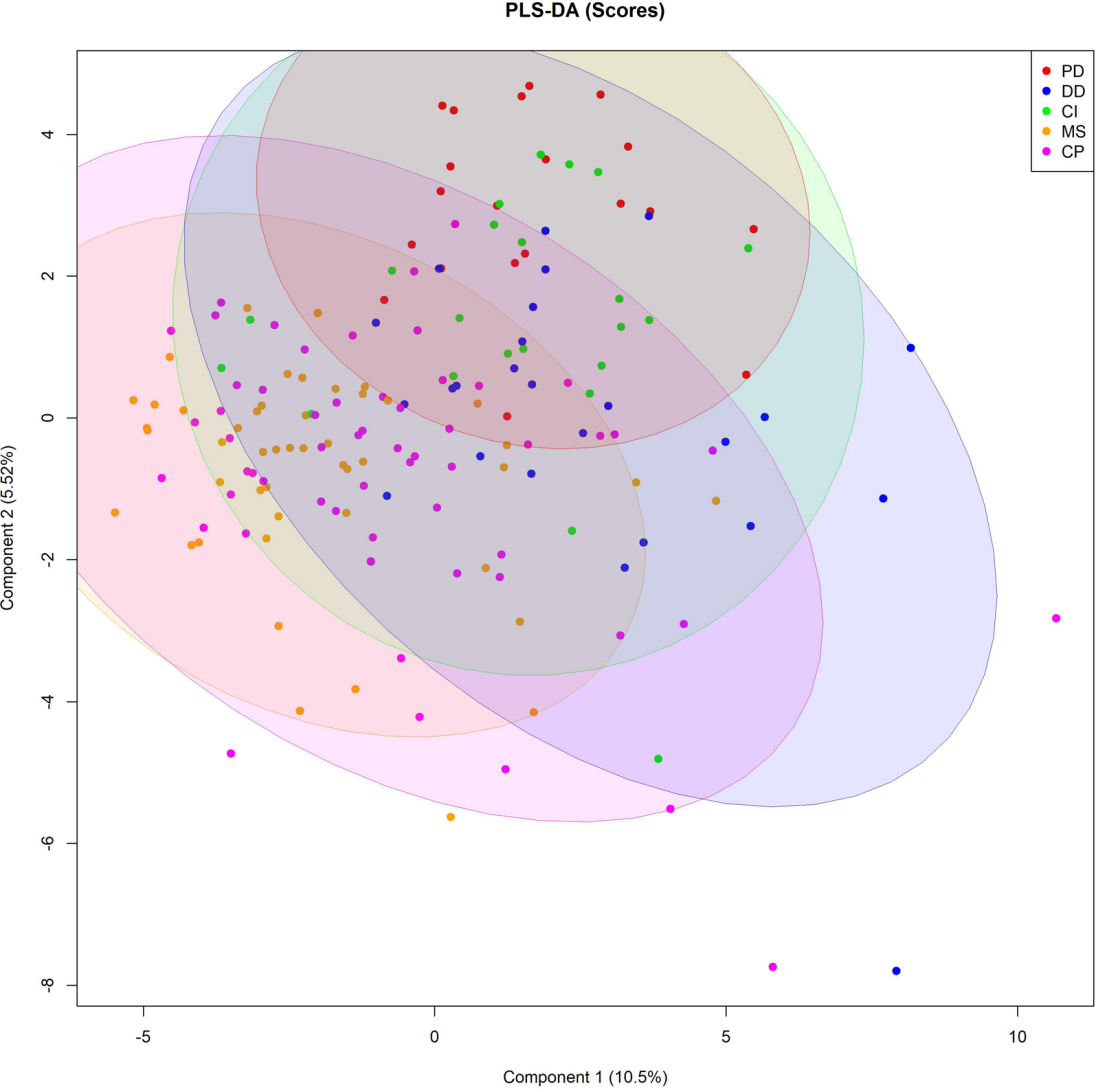
Partial least squares-discriminant analysis (PLS-DA) of 5 different disease groups. Cross-validated score plot for the comparison of 5 different patient groups for CSF and serum samples combined. Colored ellipses represent 95% confidence intervals. Colored points represent individual samples. PLS-DA scores are 10.5% for component 1 and 5.52% for component 2. *PD* Parkinson´s disease, *DD* degenerative diseases, *CI* cerebral ischemia, *MS* multiple sclerosis, *CP* control patients

We next aimed to differentiate patient groups based on absolute metabolite levels. To this end, a multivariate analysis was applied on the entire data set (CSF and serum samples combined) using a partial least squares discriminant analysis (PLS-DA). Figure 3 shows that the 5 individual groups formed clusters. In particular, MS and PD were represented in different clusters. However, clusters also overlapped to varying degrees. For instance, MS and CP were grouped closely together. The R2Y value (0.309) as well as the Q2Y value (0.12) indicated a low model accuracy and predictive performance. Hence, PLS-DA demonstrated only moderate group differentiation when considering all five patient groups at the same time.

**Fig. 4:**
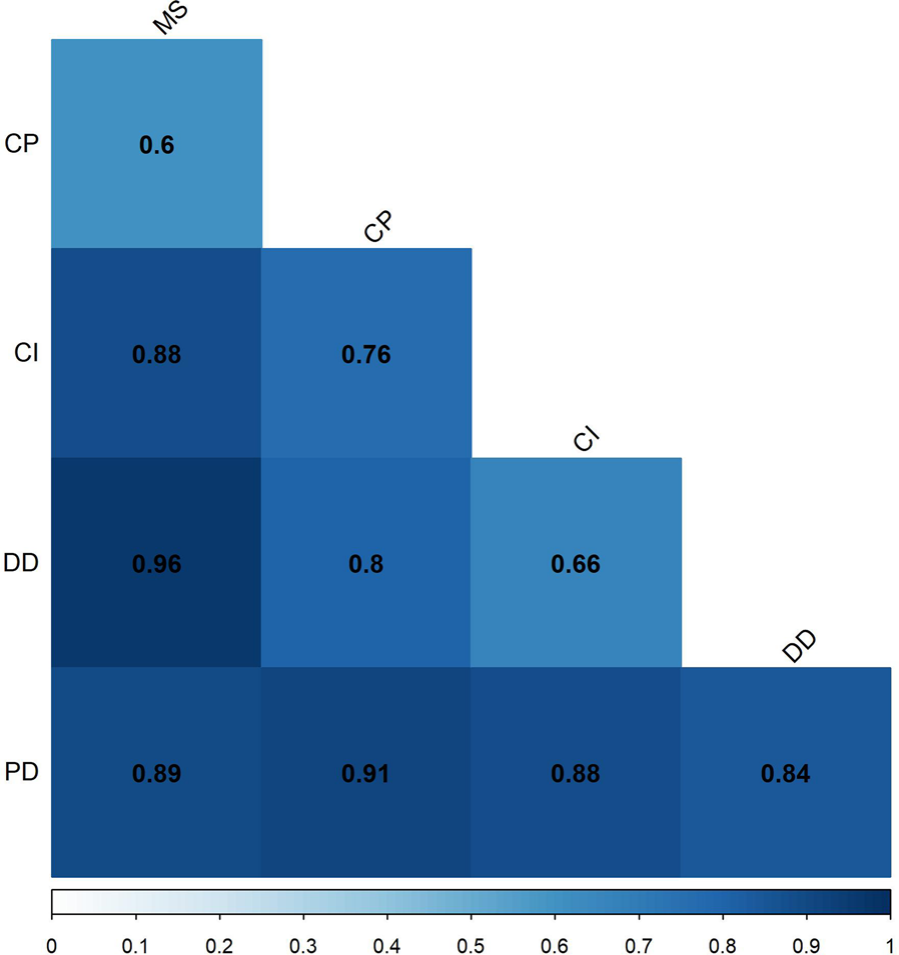
Correlation plot of AUC values from random forest model building and group comparison. Each cell in the matrix shows the correlation coefficient between two patient groups. Each row and each column represents one patient group as labelled on the left or on the top. At the intersection of rows and columns, the area under the curve (AUC) value for the random forest model between the group from the row label and the group from the column label is indicated. Legend for the color code can be found at the bottom of the plot; the darker the color, the higher the AUC value. *PD* Parkinson´s disease, *DD* degenerative diseases, *CI* cerebral ischemia, *MS* multiple sclerosis, *CP* control patients

Next, random forest statistical models were built to assess the differential power of absolute metabolite levels in CSF and serum in paired group comparisons**.** Figure 4 shows AUC values for each comparison. PD and CP (AUC = 0.91), PD and MS (AUC = 0.89), and DD and MS (AUC = 0.96) could be separated with high AUC values. Consistent with the results of PLS-DA, differentiation between MS and CP was lower (AUC = 0.6).

### Comparison of the Parkinson’s disease group with the degenerative disease group

**Fig. 5:**
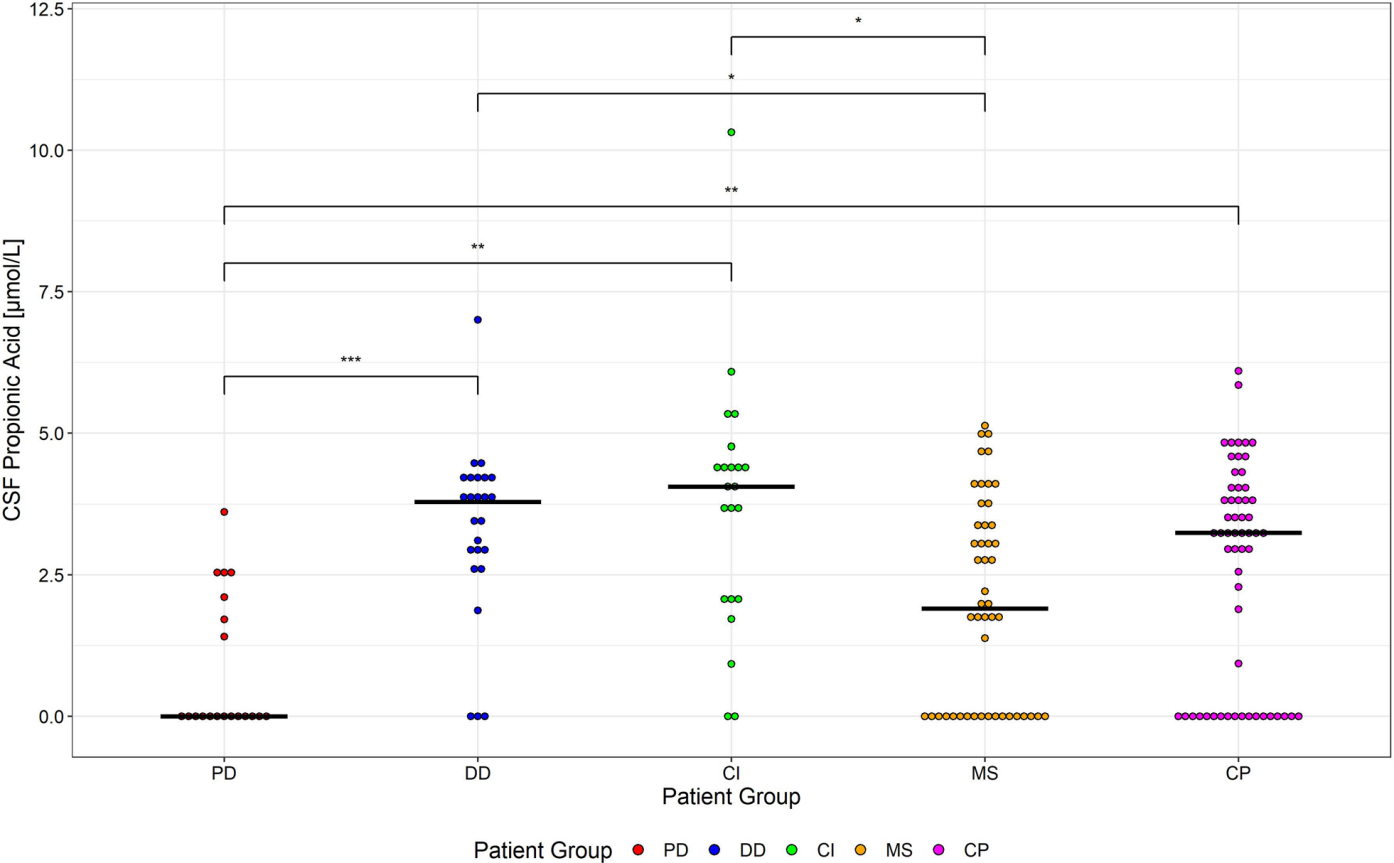
Propionic acid levels in CSF in the different groups of patients. CSF propionic acid concentration per patient group (*PD* = Parkinson´s disease (red), *DD* degenerative diseases (blue), *CI* Cerebral Ischemia (green), *MS* Multiple Sclerosis (yellow), *CP* Control Patients (pink)). Bold black line represent the median. Corrected p-values are shown in *-notation (p ≤ 0.001 (***), p ≤ 0.01 (**), p ≤ 0.05 (*)). Non-significant p-values are not shown

While clinical differentiation of PD from MS, CI, and CP is often straightforward based on clinical and radiological findings, in clinical practice, the differentiation between PD and other DD is more meaningful and can be more challenging. To explore whether metabolome profiles may aid in this differentiation, we compared metabolome profiles of patients with PD and DD in more detail. Table 2 lists all statistically significant different metabolites in the PD and DD groups. Patients with PD had significantly lower CSF propionic acid, methanol, 3-hydroxyisobutyric acid, pyruvic acid and myo-inositol levels compared to patients with DD. In contrast, PD patients had significantly higher CSF ascorbic acid and CSF and serum tyrosine levels, as well as serum ornithine levels, compared to patients with DD. The most striking difference between patients with PD and DD was an about fourfold lower level of CSF propionic acid in patients with PD (Fig. 5).

**Table 2:** Statistically significant different metabolites in patients with Parkinson’s disease (PD) and other degenerative diseases (DD) ordered by p-values

Metabolite	Matrix	PD, mean (sd)	DD, mean (sd)	p-value	Corrected p-value	Fold change	Abs. Cohen’s d	Potential Pathway(s)
Propionic acid	CSF	0.82 (1.22)	3.3 (1.56)	5.7880 × 10^–6^	***	0.25	1.74	Microbial metabolism/fatty acid metabolism
Methanol	CSF	50.85 (44.31)	105.54 (23.24)	0.0002	*	0.48	1.6	Drug metabolite, Microbial metabolism
3-Hydroxy-isobutyric acid	CSF	13.12 (2.9)	17.53 (4.45)	0.0004	*	0.75	1.15	Valine metabolism, Ketone/fatty acid metabolism
Ascorbic acid	CSF	225 (35.23)	178.54 (46.49)	0.0008	*	1.26	1.11	Antioxidant transport and/or release, Redox reaction and antioxidative effects
Pyruvic acid	CSF	95.94 (26.93)	121.04 (25.91)	0.0010	*	0.79	0.95	Energy metabolism
myo-Inositol	CSF	158.21 (30.36)	205.88 (54.34)	0.0016	*	0.77	1.05	Neuronal and glial activity
Ornithine	Serum	0.09 (0.03)	0.06 (0.02)	0.0027	*	1.41	0.93	Urea cycle
Tyrosine	CSF	12.44 (2.89)	9.72 (2.39)	0.0029	*	1.28	1.04	Precursor for neurotransmitters
Tyrosine	Serum	0.07 (0.02)	0.05 (0.01)	0.0036	*	1.36	1.11	Precursor for neurotransmitters
Creatinine	CSF	71.8 (16.5)	92.8 (28.92)	0.0039	*	0.77	0.87	Breakdown of creatine
Hypoxanthine	CSF	0.65 (1.19)	2.04 (1.73)	0.0047	*	0.32	0.92	Purine metabolism pathway
Lactic acid	CSF	1799.16 (463.61)	2092.51 (397.11)	0.0048	*	0.86	0.69	Anaerobic glycolysis pathway

*CSF* cerebrospinal fluid

Statistical significance was determined using the Wilcoxon-Mann–Whitney test. Resulting p-values were corrected for multiple testing (FDR correction with respect to all metabolites tested). The corrected p-values were translated into * notation as follows: ***p ≤ 0.001, **p ≤ 0.01, and *p ≤ 0.05. Measurement unit for CSF is µmol/L and for serum mmol/L

**Fig. 6:**
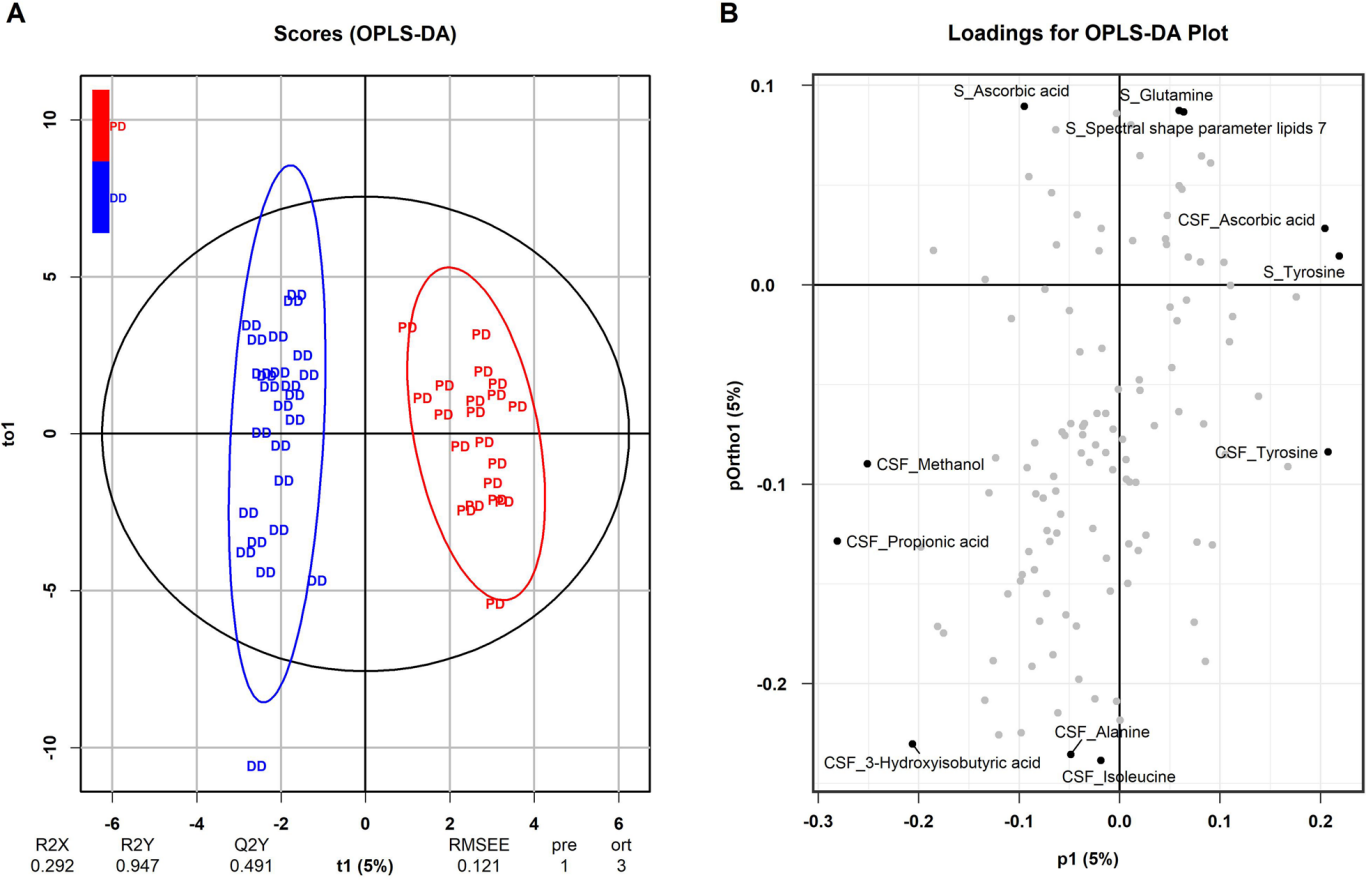
Orthogonal partial least square discriminant analysis (OPLS-DA). **A** Cross-validated score plot for the comparison of patients with PD (in red) and patients with other degenerative diseases (in blue) for CSF and serum samples combined. Colored ellipses represent 95% confidence intervals. Colored group abbreviations represent individual samples. OPLS-DA score is 5% for component 1. The R2Y value is 0.947, and the Q2Y value is 0.491. **B** Loadings for OPLS-DA plot. CSF metabolites indicated by “CSF_”, serum metabolites by “S_”

A multivariate analysis demonstrated a clear discrimination between PD and DD. As the R2Y (0.947) value indicates a high model predictive accuracy, and the Q2Y value of 0.491 indicates a good predictive quality, the OPLS-DA model is very stable and has good reliability (Fig. 6). Discriminating metabolites were very similar to the ones in the univariate analysis and included propionic acid, methanol, 3-hydroxy-isobutyric acid, ascorbic acid (CSF) and tyrosine (CSF and serum).

## Discussion

The key findings of the present study, which, to the best of our knowledge, is one of the most comprehensive analysis of metabolome profiles in CSF and serum of patients with different categories of neurological diseases conducted to date, are (i) a good agreement between CSF glucose as well as CSF lactate levels as measured by routine clinical chemistry methods and NMR spectroscopy; (ii) the lifespin^®^ metabolomic profiling platform detected a high number of different metabolites in CSF (n = 67) and serum (n = 83); (iii) an association of CSF concentrations of some metabolites with age as well as with blood–brain-barrier function; (iv) detection rates of serum and CSF metabolites were overall similar between the different patient groups; (v) based on absolute metabolite levels in CSF and serum, two group comparisons permitted differentiation between some patient groups with high AUC values; (vi) orthogonal partial least square discriminant analysis using absolute metabolite levels in CSF and serum permitted separation of PD and DD.

The strong agreement between CSF glucose as well as CSF lactate levels as measured by routine clinical chemistry and NMR spectroscopy indicates that NMR spectroscopy is an appropriate method for quantification of metabolites in CSF.

The comparative analyses of contemporaneously withdrawn CSF and serum samples contributes to the definition of the landscape of the CSF and serum metabolome in patients with different neurological diseases. The predominant detection of some metabolites in CSF, such as acetylcholine, a neurotransmitter, or 2-hydroxybutyric acid, which is related to energy metabolism, is consistent with the concept that CNS metabolites accumulate in the CSF, where they can be measured and where their concentrations might reflect physiological or pathological processes.

The exclusive detection of some metabolites in either serum or CSF has important implications for further usage of such parameters as potential biomarkers for neurological diseases. Metabolites only present in serum are unlikely to reflect disease processes in the CNS. Conversely, usage of metabolites exclusively present in CSF is limited by the fact that they may only be investigated invasively through lumbar punctures. Therefore, the most interesting metabolites in terms of potential disease biomarkers might be those that originate in the CNS and subsequently reach the systemic circulation, similar to neurofilament light chain protein, a protein biomarker of neuroaxonal injury [7]

The association of several metabolites, in particular, metabolites in CSF, with age highlights that age needs to be taken into account when analysing such metabolites as potential biomarkers for neurological diseases.

The increase of CSF levels of certain metabolites with increasing Q_alb_, a marker of declining blood–brain-barrier function, suggests that those metabolites could enter the CNS by passive diffusion across the blood–brain-barrier [8]. In contrast, metabolites, which show no dependency on blood–brain barrier function may reach the CNS by transporters [6]. Nevertheless, the clarification of the precise mechanisms of entry of metabolites into the CNS and the precise origin of the net concentrations of metabolites in CSF remain to be further studied and are beyond the scope of the present work.

The overall similar detection rates of the different metabolites in CSF and serum among the 5 different groups of patients do not suggest that there may be individual or unique metabolites associated with any one the analysed disease categories.

However, a multivariate analysis (PLS-DA) and model building (random forest) revealed that the assessment of the entire metabolite profile in CSF and serum, rather than focussing on one single parameter, could assist in the differentiation of categories of neurological diseases. While the PLS-DA showed some overlap between the 5 disease groups analysed in this work, high AUC values could be observed in two-group comparisons, especially between MS/DD and MS/PD, by using the random forest algorithm.

Whereas differentiation between MS/DD and MS/PD is usually straightforward on clinical grounds, the differentiation between PD and other DD can be more challenging and represents a relevant medical need. We therefore specifically addressed whether metabolite profiles might aid in the differentiation of PD and DD.

We identified significantly elevated ascorbic acid in CSF and tyrosine levels in CSF and serum and ornithine levels in serum as well as significantly decreased levels of propionic acid, methanol, 3-hydroxyisobutyric acid, pyruvic acid and myo-inositol in CSF in PD compared to DD.

Accordingly, orthogonal partial least square discriminant analysis using absolute metabolite levels in CSF and serum permitted separation of PD and DD. These results suggest that there are distinguishing differences in the metabolic profile of these two groups.

The most prominent difference between PD and DD was a fourfold decrease in the levels of propionic acid in CSF of patients with PD. Propionic acid is a short-chain fatty acid derived from microbial fermentation of dietary substrates by colonic bacteria [12]. In an in vitro model of PD using cultured primary mesencephalic cells treated with the pesticide rotenone, which induces dopaminergic cell damage, propionic acid promoted cell survival of rotenone-treated dopaminergic cells [14].The reduced levels of propionic acid in CSF observed in the present study therefore appear of interest as they might hypothetically contribute to reduced protection of dopaminergic neurons in patients with PD. While we are not aware of any previous studies analysing propionic acid levels in CSF of patients with PD, future studies of the levels of propionic acid in CSF of patients with PD and the possible role of propionic acid in PD appear warranted.

Elevated tyrosine levels, a precursor molecule of dopamine, in PD may be attributed to feedback mechanisms resulting from dopamine deficiency. This hypothesis is supported by previous metabolomic assessments using NMR spectroscopy, which consistently demonstrated an increase in tyrosine concentrations in the CSF of individuals with PD [5]. One proposed mechanism for this observation involves α-synuclein-mediated activation of glycolysis, leading to an upregulation of the tyrosine synthesis pathway. This metabolomic pathway, when dysregulated, can lead to an accumulation of tyrosine. Additionally, this metabolomic dysregulation has been linked to oxidative stress and cell death through the accumulation of citrate and acetate [11]. Further supporting evidence comes from studies examining saliva samples of patients with PD, which also found elevated tyrosine levels compared to controls [9]. Altogether, our findings strengthen the association between altered tyrosine metabolism and PD.

Nevertheless, other metabolites found to be differentially abundant in CSF of PD and DD in our work have previously not consistently been associated with PD, which could be related to differences in the applied methodologies and in the comparator groups [1, 10, 18, 21, 24]. While quinolinic acid has previously been implicated in PD [1], quinolinic acid was not detected by the methodology applied in this work and could therefore not be assessed in the present study.

Of note, as all PD patients included in this work were treated with dopaminergic therapy, we cannot exclude that the observed effects may be attributable to therapy rather than the disease itself. Future studies should thus analyse the metabolome of untreated PD patients.

Strengths of this study include the highly standardized collection and storage of CSF and serum samples from clinically well-characterized groups of patients as well as the highly standardized measurement of metabolites in CSF and serum using an established metabolomic profiling platform.

Among the limitations of this study are the relatively lower patient numbers in the CI, PD and DD compared to the MS and CP groups. In addition, diagnoses of patients included in the DD and CP group were rather heterogeneous. We cannot exclude that this may have influenced the findings in these groups. Still, as none of the patients in the CP group had signs of inflammation in the CSF, any underlying pathology in the CP group was below the threshold of producing inflammatory changes in the CSF. Furthermore, as some of the patients studied in this work were not therapy-naïve, we cannot exclude a potential confounding influence of medications, e.g. intrathecal corticosteroids or dopaminergic therapy, on metabolite profiles. Finally, this study did not include independent validation cohorts. Future studies may therefore aim to reproduce the present findings in independent patient cohorts with a particular focus on the comparison of PD and DD. Furthermore, as analyses of the associations of metabolites with disease severity or activity were beyond the scope of the present work, future studies may also address this issue.

## Conclusions

Altogether, the present study demonstrates that NMR spectroscopy is a suitable method for quantification of metabolites in CSF and serum and thus for gaining insights into metabolic alterations in neurological diseases. Our findings outline the landscape of the CSF and serum metabolome in different categories of neurological diseases and identify age and blood–brain-barrier function as relevant co-factors for CSF levels of certain metabolites. Metabolome profiles as determined by NMR spectroscopy may potentially aid in differentiating groups of patients with different neurological diseases, including clinically meaningful differentiations, such as PD from other DD.

## Supplementary Information

Supplementary Material 1.

## Data Availability

All datasets generated in the current study are included in the manuscript and the Supplementary Material.

## Abbreviations

BBB: Blood Brain Barrier
CI: Cerebral Ischemia
CSF: Cerebrospinal fluid
DD: Degenerative Diseases
DE: Dementia
LED: Levodopa Equivalent Dose
MDS: The international parkinson and movement disorder society
MMST: Mini mental status test
mRS: Modified Ranking Scale
MS: Multiple sclerosis
MRS: Magnetic resonance spectroscopy
NIHSS: National institutes of health stroke scale
NMDA: N-methyl-D-aspartate
NMR: Nuclear magnetic resonance
PD: Parkinson’s Disease
PPMS: Primary progressive multiple sclerosis
Q_alb_: CSF/serum albumin quotient
RRMS: Relapsing remitting multiple sclerosis
SCFA: Short-chain fatty acids
UPDRS: Unified Parkinson’s disease rating scale
