# Supplementary material for: Comprehensive analysis of the cerebrospinal fluid and serum metabolome in neurological diseases
Source: J Neuroinflammation. 2024 Sep 26;21:234. doi: 10.1186/s12974-024-03218-0 (PMC11430517; doi:10.1186/s12974-024-03218-0)
Supplement: Supplementary file 1 — Supplementary Material 1. [file 12974_2024_3218_MOESM1_ESM.docx]

# **Supplementary material**

**Table S1. Metabolites detected in CSF and serum, CSF only, or serum only by NMR spectroscopy**

| **51 metabolites detected**  **in CSF and serum** | | | | **16 metabolites detected**  **in CSF only** | **32 metabolites detected**  **in serum only** |
| --- | --- | --- | --- | --- | --- |
| 1,2-Propanediol | Citric acid | Isopropanol | Tyrosine | 1,7-Dimethyluric acid | 2 Spectral shape parameter fatty acids (s, u)^a^ |
| 2,3-Butanediol | Creatine | Lactic acid | Urea | 2-Hydroxyisovaleric acid | Dimethylglycine |
| 2-Hydroxybutyric acid | Creatinine | Leucine | Valine | 3-Hydroxyisobutyric acid | Dimethylmalonic acid |
| 2-Hydroxyisobutyric acid | Dimethylamine | Lysine |  | 3-Hydroxyisovaleric acid | Glucuronic acid |
| 3-Hydroxybutyric acid | Dimethylsulfone | Mannose |  | Acetoin | 2 Glycoprotein acetyls (A and B) |
| Acetic acid | Ethanol | Methanol |  | Acetylcholine | Mannitol |
| Acetoacetic acid | Ethanolamine | Methionine |  | Butyric acid | Phosphatidylcholine |
| Acetone | Formic acid | myo-Inositol |  | Carnitine | Sarcosine |
| Adenine | Glucose | Ornithine |  | Fructose | 16 Spectral shape parameter lipids (1–16)^b^ |
| Alanine | Glutamic acid | Phenylalanine |  | Fumaric acid | Sphingomyeline |
| Albumin | Glutamine | Proline |  | Galactose | Succinic acid |
| Arginine | Glycerol | Propanol |  | Inosine | Total cholesterol |
| Ascorbic acid | Glycine | Propionic acid |  | Isobutyric acid | Total fatty acids |
| Asparagine | Histidine | Pyruvic acid |  | Ketovaline | Total protein |
| Aspartic acid | Hypoxanthine | Serine |  | Pyroglutamic acid | Total triglycerides |
| Choline | Isoleucine | Threonine |  | tert-Butanol |  |

^a^Spectral shape parameter fatty acids describe protons in a fatty acid chain and exist in saturated (s) and unsaturated (u) constitution.

^b^Spectral shape parameter lipids capture the NMR detectable cholesterol in lipoproteins.

CSF = cerebrospinal fluid.

**Table S2. Association of storage times with metabolite concentrations in serum and CSF**

| **Metabolite** | **material** | ***r* value** | ***p* value** | **corrected *p* value** | **Number of pairs** |
| --- | --- | --- | --- | --- | --- |
| 2-Hydroxybutyric acid | serum | 0.4 | 0.03 | n.s. | 28 |
| Acetoacetic acid | serum | -0.2 | 0.02 | n.s. | 168 |
| Acetone | serum | -0.2 | 0.04 | n.s. | 173 |
| Choline | serum | -0.2 | 0.02 | n.s. | 170 |
| Citric acid | serum | -0.2 | 0.004 | n.s. | 173 |
| Formic acid | serum | -0.2 | 0.03 | n.s. | 171 |
| Hypoxanthine | serum | 0.5 | 0.04 | n.s. | 17 |
| Leucine | serum | -0.2 | 0.048 | n.s. | 173 |
| Methanol | CSF | -0.2 | 0.023 | n.s. | 173 |
| Albumin | serum | 0.2 | 0.004 | n.s. | 173 |
| Total Protein | serum | 0.2 | 0.019 | n.s. | 173 |
| Cholesterol Sub19 | serum | 0.2 | 0.024 | n.s. | 172 |

n.s. = not significant

The median (minimum - maximum) storage time, i.e. the interval between CSF/serum withdrawal and NMR spectroscopy analysis, of the CSF/serum samples from the 173 patients analyzed in this study was 10 (0 - 34) months. To analyze a potential influence of storage times on metabolite concentrations, we analyzed the association of either CSF or serum concentrations of all 84 metabolites detected in CSF, serum, or both with storage times by Spearman rank correlations using data of all patients (n=173) included in this study. As shown in the Table S2, concentrations of 12 metabolites (11 in serum, 1 in CSF) seemed be to be either positively or negatively associated (uncorrected *p*<0.05) with storage times. However, corresponding *r* values were low, indicating very weak associations. Furthermore, following correction for multiple testing by the Bonferroni method, i.e. by multiplying the uncorrected *p* values by the number of statistical tests performed in this analysis (n = 132), none of the resulting corrected *p* values remained significant. Altogether, these data suggest that storage times of up to 34 months did overall not have a relevant influence on the serum or CSF concentrations of the metabolites analyzed in this work.

**Table S3. Association of metabolites in CSF or serum with age**

| **Metabolite** | **material** | ***p* value** | **r value** | **Number of pairs** |
| --- | --- | --- | --- | --- |
| 2-Hydroxyisobutyric acid | CSF | <0.0001 | 0.6 | 42 |
| 3-Hydroxyisobutyric acid | CSF | <0.001 | 0.4 | 58 |
| 3-Hydroxyisovaleric acid | CSF | <0.001 | 0.4 | 58 |
| Acetoacetic acid | CSF | 0.0045 | 0.3 | 58 |
| Alanine | CSF | <0.0001 | 0.5 | 58 |
| Albumin | serum | 0.043 | -0.3 | 58 |
| Albumin | CSF | <0.001 | 0.5 | 58 |
| Choline | serum | <0.0001 | 0.5 | 57 |
| Choline | CSF | <0.0001 | 0.5 | 58 |
| Citric acid | serum | 0.039 | -0.3 | 58 |
| Citric acid | CSF | <0.0001 | 0.5 | 58 |
| Creatine | CSF | 0.009 | 0.3 | 58 |
| Creatinine | CSF | <0.0001 | 0.6 | 58 |
| Dimethylamine | CSF | <0.0001 | 0.5 | 58 |
| Formic acid | CSF | 0.017 | 0.3 | 58 |
| Glutamine | CSF | 0.023 | 0.3 | 58 |
| Glycine | CSF | 0.007 | 0.5 | 26 |
| Isoleucine | serum | 0.008 | 0.3 | 58 |
| Lactic acid | CSF | 0.002 | 0.4 | 58 |
| Leucine | CSF | 0.001 | 0.4 | 58 |
| Mannose | serum | 0.009 | 0.4 | 44 |
| Myo-Inositol | CSF | 0.026 | 0.3 | 58 |
| Ornithine | CSF | 0.004 | 0.4 | 51 |
| Phenylalanine | serum | 0.042 | 0.3 | 54 |
| Proline | serum | 0.019 | 0.3 | 58 |
| Pyruvic acid | CSF | 0.011 | 0.33 | 58 |
| Sarcosine | serum | 0.018 | 0.3 | 48 |
| Spectral shape parameter fatty acids (saturated) ^a^ | serum | 0.034 | 0.3 | 58 |
| Spectral shape parameter fatty acids (unsaturated) ^a^ | serum | 0.046 | 0.3 | 58 |
| Threonine | serum | 0.035 | -0.3 | 57 |
| Tyrosine | CSF | <0.001 | 0.5 | 54 |
| Urea | serum | 0.015 | 0.3 | 58 |
| Urea | CSF | <0.001 | 0.5 | 58 |
| Valine | CSF | <0.001 | 0.5 | 58 |

The association of the CSF or serum concentrations of all 84 metabolites detected in CSF, serum, or both with age was assessed in 58 control patients by Spearman rank correlations. The table lists all metabolites with significant (*p*<0.05) associations with age.

^a^Spectral shape parameter fatty acids describe protons in a fatty acid chain and exist in saturated (s) and unsaturated (u) constitution.

CSF = cerebrospinal fluid.

**Table S4. Association of CSF/serum metabolite quotients with the CSF/serum albumin quotient (Q_Alb_)**

| **Metabolite (CSF/serum quotient)** | ***p* value** | **r value** | **Number of pairs** |
| --- | --- | --- | --- |
| *2-Hydroxyisobutyric acid* | 0.014 | 0.5 | 21 |
| *Alanine* | <0.0001 | 0.5 | 58 |
| Albumin | <0.001 | 0.8 | 58 |
| *Citric acid* | 0.002 | 0.4 | 58 |
| Glucose | 0.035 | 0.3 | 58 |
| *Glutamine* | <0.001 | 0.7 | 58 |
| *Glycine* | <0.001 | 0.7 | 26 |
| Histidine | 0.013 | 0.3 | 57 |
| Isoleucine | <0.0001 | 0.5 | 58 |
| *Lactic acid* | 0.044 | 0.3 | 58 |
| *Leucine* | 0.006 | 0.4 | 58 |
| Methanol | <0.001 | 0.5 | 58 |
| *Ornithine* | 0.005 | 0.4 | 51 |
| Propanol | <0.001 | 0.8 | 16 |
| Threonine | <0.001 | 0.4 | 55 |
| *Tyrosine* | <0.001 | 0.6 | 51 |
| *Urea* | 0.006 | 0.4 | 58 |
| *Valine* | <0.001 | 0.5 | 58 |

The association of the CSF/serum quotient of 48 metabolites detected in both CSF and serum with the albumin CSF/serum quotient (Q_alb_) was assessed in 58 control patients by Spearman rank correlations. The table lists all CSF/serum metabolite quotients with significant (*p*<0.05) associations with Q_alb_. Metabolites whose CSF levels increased with increasing age (see Table S2) are shown in italics.

CSF = cerebrospinal fluid.

**Table S5. Detection rates of 48 metabolites detected in both CSF and serum among the 5 different groups of patients.**

|  | **Parkinson’s Disease**  **(n = 20)** | | **Degenerative Diseases**  **(n = 25)** | | **Cerebral Ischemia**  **(n = 22)** | | **Multiple Sclerosis**  **(n = 48)** | | **Control patients**  **(n = 58)** | |
| --- | --- | --- | --- | --- | --- | --- | --- | --- | --- | --- |
| **Metabolite** | **Serum** | **CSF** | **Serum** | **CSF** | **Serum** | **CSF** | **Serum** | **CSF** | **Serum** | **CSF** |
| 1,2-Propanediol | 2 (10%) | 19 (95%) | 2 (8%) | 22 (88%) | 8 (36%) | 18 (82%) | 9 (19%) | 37 (77%) | 12 (21%) | 51 (88%) |
| 2,3-Butanediol | 2 (10%) | 19 (95%) | 0 (0%) | 22 (88%) | 1 (5%) | 18 (82%) | 1 (2%) | 37 (77%) | 1 (2%) | 51 (88%) |
| 2-Hydroxybutyric acid | 4 (20%) | **20 (100%)** | 2 (8%) | **25 (100%)** | 2 (9%) | **22 (100%)** | 8 (17%) | **48 (100%)** | 12 (21%) | **58 (100%)** |
| 2-Hydroxyisobutyric acid | 10 (50%) | 17 (85%) | 13 (52%) | **25 (100%)** | 9 (41%) | 20 (91%) | 24 (50%) | 34 (71%) | 28 (48%) | 42 (72%) |
| 3-Hydroxybutyric acid | **20 (100%)** | **20 (100%)** | 23 (92%) | 24 (96%) | **22 (100%)** | 21 (95%) | **48 (100%)** | 44 (92%) | **58 (100%)** | 54 (93%) |
| Acetoacetic acid | **20 (100%)** | **20 (100%)** | **25 (100%)** | **25 (100%)** | **22 (100%)** | **22 (100%)** | 46 (96%) | **48 (100%)** | 55 (95%) | **58 (100%)** |
| Acetone | **20 (100%)** | **20 (100%)** | **25 (100%)** | **25 (100%)** | **22 (100%)** | **22 (100%)** | **48 (100%)** | **48 (100%)** | **58 (100%)** | **58 (100%)** |
| Adenine | 0 (0%) | 3 (15%) | 1 (4%) | 8 (32%) | 0 (0%) | 2 (9%) | 0 (0%) | 8 (17%) | 5 (9%) | 10 (17%) |
| Alanine | **20 (100%)** | **20 (100%)** | **25 (100%)** | **25 (100%)** | **22 (100%)** | **22 (100%)** | **48 (100%)** | **48 (100%)** | **58 (100%)** | **58 (100%)** |
| Albumin | **20 (100%)** | **20 (100%)** | **25 (100%)** | **25 (100%)** | **22 (100%)** | **22 (100%)** | **48 (100%)** | **48 (100%)** | **58 (100%)** | **58 (100%)** |
| Arginine | 17 (85%) | 10 (50%) | 23 (92%) | 6 (24%) | 20 (91%) | 10 (45%) | 39 (81%) | 13 (27%) | 52 (90%) | 17 (29%) |
| Ascorbic acid | 8 (40%) | **20 (100%)** | 15 (60%) | **25 (100%)** | 8 (36%) | **22 (100%)** | 38 (79%) | **48 (100%)** | 42 (72%) | **58 (100%)** |
| Asparagine | 13 (65%) | 8 (40%) | 18 (72%) | 2 (8%) | 16 (73%) | 1 (5%) | 39 (81%) | 9 (19%) | 49 (84%) | 5 (9%) |
| Aspartic acid | 6 (30%) | 1 (5%) | 9 (36%) | 0 (0%) | 8 (36%) | 2 (9%) | 15 (31%) | 0 (0%) | 19 (33%) | 0 (0%) |
| Choline | **20 (100%)** | **20 (100%)** | **25 (100%)** | **25 (100%)** | **22 (100%)** | **22 (100%)** | **46 (96%)** | **48 (100%)** | 57 (98%) | **58 (100%)** |
| Citric acid | **20 (100%)** | **20 (100%)** | **25 (100%)** | **25 (100%)** | **22 (100%)** | **22 (100%)** | **48 (100%)** | **48 (100%)** | **58 (100%)** | **58 (100%)** |
| Creatine | **20 (100%)** | **20 (100%)** | **25 (100%)** | **25 (100%)** | **22 (100%)** | **22 (100%)** | **48 (100%)** | **48 (100%)** | **58 (100%)** | **58 (100%)** |
| Creatinine | **20 (100%)** | **20 (100%)** | **25 (100%)** | **25 (100%)** | **22 (100%)** | **22 (100%)** | **48 (100%)** | **48 (100%)** | **58 (100%)** | **58 (100%)** |
| Dimethylamine | 15 (75%) | **20 (100%)** | 22 (88%) | **25 (100%)** | 18 (82%) | **22 (100%)** | 30 (62%) | **48 (100%)** | 38 (66%) | **58 (100%)** |
| Dimethylsulfone | **20 (100%)** | **20 (100%)** | **25 (100%)** | **25 (100%)** | **22 (100%)** | **22 (100%)** | **48 (100%)** | **48 (100%)** | **58 (100%)** | **58 (100%)** |
| Ethanolamine | 8 (40%) | 19 (95%) | 10 (40%) | **25 (100%)** | 4 (18%) | **22 (100%)** | 20 (42%) | **48 (100%)** | 28 (48%) | **58 (100%)** |
| Formic acid | 19 (95%) | **20 (100%)** | 24 (96%) | **25 (100%)** | **22 (100%)** | **22 (100%)** | **48 (100%)** | **48 (100%)** | **58 (100%)** | **58 (100%)** |
| Glucose | **20 (100%)** | **20 (100%)** | **25 (100%)** | **25 (100%)** | **22 (100%)** | **22 (100%)** | **48 (100%)** | **48 (100%)** | **58 (100%)** | **58 (100%)** |
| Glutamic acid | **20 (100%)** | 1 (5%) | **25 (100%)** | 1 (4%) | **22 (100%)** | 1 (5%) | **48 (100%)** | 1 (2%) | **58 (100%)** | 1 (2%) |
| Glutamine | **20 (100%)** | **20 (100%)** | **25 (100%)** | **25 (100%)** | **22 (100%)** | **22 (100%)** | **48 (100%)** | **48 (100%)** | **58 (100%)** | **58 (100%)** |
| Glycerol | 18 (90%) | **20 (100%)** | 22 (88%) | 23 (92%) | 18 (82%) | 20 (91%) | 43 (90%) | 37 (77%) | 51 (88%) | 51 (88%) |
| Glycine | **20 (100%)** | 17 (85%) | **25 (100%)** | 22 (88%) | **22 (100%)** | 13 (59%) | **48 (100%)** | 19 (40%) | **58 (100%)** | 26 (45%) |
| Histidine | **20 (100%)** | **20 (100%)** | **25 (100%)** | **25 (100%)** | **22 (100%)** | **22 (100%)** | **48 (100%)** | 47 (98%) | **58 (100%)** | 57 (98%) |
| Hypoxanthine | 2 (10%) | 5 (25%) | 4 (16%) | 16 (64%) | 2 (9%) | 10 (45%) | 3 (6%) | 25 (52%) | 6 (10%) | 28 (48%) |
| Isoleucine | **20 (100%)** | **20 (100%)** | **25 (100%)** | **25 (100%)** | **22 (100%)** | **22 (100%)** | **48 (100%)** | 47 (98%) | **58 (100%)** | **58 (100%)** |
| Lactic acid | **20 (100%)** | **20 (100%)** | **25 (100%)** | **25 (100%)** | **22 (100%)** | **22 (100%)** | **48 (100%)** | **48 (100%)** | **58 (100%)** | **58 (100%)** |
| Leucine | **20 (100%)** | **20 (100%)** | **25 (100%)** | **25 (100%)** | **22 (100%)** | **22 (100%)** | **48 (100%)** | **48 (100%)** | **58 (100%)** | **58 (100%)** |
| Lysine | **20 (100%)** | **20 (100%)** | **25 (100%)** | **25 (100%)** | **22 (100%)** | **22 (100%)** | **48 (100%)** | **48 (100%)** | **58 (100%)** | **58 (100%)** |
| Mannose | 19 (95%) | **20 (100%)** | 24 (96%) | **25 (100%)** | **22 (100%)** | **22 (100%)** | 42 (88%) | **48 (100%)** | 44 (76%) | **58 (100%)** |
| Methanol | **20 (100%)** | **20 (100%)** | **25 (100%)** | **25 (100%)** | **22 (100%)** | **22 (100%)** | **48 (100%)** | **48 (100%)** | **58 (100%)** | **58 (100%)** |
| Methionine | 19 (95%) | 0 (0%) | 23 (92%) | 1 (4%) | **22 (100%)** | 0 (0%) | **48 (100%)** | 8 (17%) | 56 (97%) | 8 (14%) |
| myo-Inositol | 17 (85%) | **20 (100%)** | 23 (92%) | **25 (100%)** | 21 (95%) | **22 (100%)** | 42 (88%) | **48 (100%)** | 48 (83%) | **58 (100%)** |
| Ornithine | **20 (100%)** | 19 (95%) | **25 (100%)** | **25 (100%)** | **22 (100%)** | 18 (82%) | **48 (100%)** | 41 (85%) | **58 (100%)** | 51 (88%) |
| Phenylalanine | 19 (95%) | **20 (100%)** | **25 (100%)** | 23 (92%) | **22 (100%)** | 20 (91%) | 45 (94%) | 41 (85%) | 54 (93%) | 51 (88%) |
| Proline | **20 (100%)** | 1 (5%) | **25 (100%)** | 1 (4%) | **22 (100%)** | 1 (5%) | **48 (100%)** | 4 (8%) | **58 (100%)** | 1 (2%) |
| Propanol | 3 (15%) | 15 (75%) | 6 (24%) | 22 (88%) | 8 (36%) | 18 (82%) | 19 (40%) | 28 (58%) | 19 (33%) | 42 (72%) |
| Propionic acid | 2 (10%) | 7 (35%) | 3 (12%) | 22 (88%) | 1 (5%) | 20 (91%) | 4 (8%) | 30 (62%) | 5 (9%) | 40 (69%) |
| Pyruvic acid | **20 (100%)** | **20 (100%)** | **25 (100%)** | **25 (100%)** | **22 (100%)** | **22 (100%)** | **48 (100%)** | **48 (100%)** | **58 (100%)** | **58 (100%)** |
| Serine | **20 (100%)** | 1 (5%) | **25 (100%)** | 1 (4%) | **22 (100%)** | 1 (5%) | **48 (100%)** | 6 (12%) | **58 (100%)** | 4 (7%) |
| Threonine | **20 (100%)** | **20 (100%)** | **25 (100%)** | **25 (100%)** | **22 (100%)** | 20 (91%) | **48 (100%)** | **48 (100%)** | 57 (98%) | 56 (97%) |
| Tyrosine | **20 (100%)** | **20 (100%)** | **25 (100%)** | **25 (100%)** | **22 (100%)** | **22 (100%)** | 45 (94%) | 39 (81%) | 55 (95%) | 54 (93%) |
| Urea | **20 (100%)** | **20 (100%)** | **25 (100%)** | **25 (100%)** | **22 (100%)** | **22 (100%)** | **48 (100%)** | **48 (100%)** | **58 (100%)** | **58 (100%)** |
| Valine | **20 (100%)** | **20 (100%)** | **25 (100%)** | **25 (100%)** | **22 (100%)** | **22 (100%)** | **48 (100%)** | **48 (100%)** | **58 (100%)** | **58 (100%)** |

Metabolites that could be detected in all patients of a patient group (100% detection rate) are indicated in bold.

Acetic acid, ethanol and isopropanol are not listed here as they were excluded from the statistical analysis (see main text).

CSF = cerebrospinal fluid.

**Table S6. Detection rates of 20 metabolites detected in serum only.**

| **Metabolite** | **Parkinson’s Disease (n = 20)** | **Degenerative Diseases (n = 25)** | **Cerebral Ischemia**  **(n = 22)** | **Multiple Sclerosis**  **(n = 48)** | **Control patients**  **(n = 58)** |
| --- | --- | --- | --- | --- | --- |
| Spectral shape parameter fatty acids (see below^a^ | **20 (100%)** | **25 (100%)** | **22 (100%)** | **48 (100%)** | **58 (100%)** |
| Dimethylglycine | 18 (90%) | 24 (96%) | 21 (95%) | 36 (75%) | 51 (88%) |
| Dimethylmalonic acid | 16 (80%) | 19 (76%) | 16 (73%) | 42 (88%) | 53 (91%) |
| Glucuronic acid | 2 (10%) | 3 (12%) | 3 (14%) | 6 (12%) | 8 (14%) |
| Glycoprotein acetyls (A and B) | **20 (100%)** | **25 (100%)** | **22 (100%)** | **48 (100%)** | **58 (100%)** |
| Mannitol | 0 (0%) | 0 (0%) | 0 (0%) | 1 (2%) | 2 (3%) |
| Phosphatidylcholine | **20 (100%)** | **25 (100%)** | **22 (100%)** | **48 (100%)** | **58 (100%)** |
| Sarcosine | 17 (85%) | 21 (84%) | 20 (91%) | 36 (75%) | 48 (83%) |
| Sphingomyeline | **20 (100%)** | **25 (100%)** | **22 (100%)** | **48 (100%)** | **58 (100%)** |
| Succinic acid | 8 (40%) | 9 (36%) | 5 (23%) | 15 (31%) | 21 (36%) |
| Total Cholesterol | **20 (100%)** | **25 (100%)** | **22 (100%)** | **48 (100%)** | **58 (100%)** |
| Spectral shape parameters lipids (1, 7, 12)^b^ | **20 (100%)** | **25 (100%)** | **22 (100%)** | **48 (100%)** | **58 (100%)** |
| Spectral shape parameters lipids (16) | **20 (100%)** | 24 (96%) | **22 (100%)** | **48 (100%)** | **58 (100%)** |
| Total fatty acids | **20 (100%)** | **25 (100%)** | **22 (100%)** | **48 (100%)** | **58 (100%)** |
| Total protein | **20 (100%)** | **25 (100%)** | **22 (100%)** | **48 (100%)** | **58 (100%)** |
| Total triglycerides | **20 (100%)** | **25 (100%)** | **22 (100%)** | **48 (100%)** | **58 (100%)** |

^a^Spectral shape parameter fatty acids describe protons in a fatty acid chain and exist in saturated (s) and unsaturated (u) constitution.

^b^Spectral shape parameter lipids capture the NMR detectable cholesterol in lipoproteins.

Metabolites that could be detected in all patients of a patient group (100% detection rate) are indicated in bold.

Spectral shape parameter lipids #2, 3, 4, 5, 6, 8, 9, 10, 11, 13, 14 and 15 are not listed here as they were excluded from the statistical analysis (see main text).

**Table S7. Detection rates of 16 metabolites detected in cerebrospinal fluid only.**

| **Metabolite** | **Parkinson’s Disease (n = 20)** | **Degenerative Diseases**  **(n = 25)** | **Cerebral Ischemia**  **(n = 22)** | **Multiple Sclerosis**  **(n = 48)** | **Control patients**  **(n = 58)** |
| --- | --- | --- | --- | --- | --- |
| 1,7-Dimethyluric acid | 18 (90%) | 16 (64%) | 18 (82%) | 42 (88%) | 51 (88%) |
| 2-Hydroxyisovaleric acid | **20 (100%)** | **25 (100%)** | **22 (100%)** | **48 (100%)** | **58 (100%)** |
| 3-Hydroxyisobutyric acid | **20 (100%)** | **25 (100%)** | **22 (100%)** | **48 (100%)** | **58 (100%)** |
| 3-Hydroxyisovaleric acid | **20 (100%)** | **25 (100%)** | **22 (100%)** | **48 (100%)** | **58 (100%)** |
| Acetoin | 3 (15%) | 0 (0%) | 1 (5%) | 0 (0%) | 1 (2%) |
| Acetylcholine | **20 (100%)** | 24 (96%) | 20 (91%) | **48 (100%)** | 56 (97%) |
| Butyric acid | 13 (65%) | 18 (72%) | 13 (59%) | 23 (48%) | 32 (55%) |
| Carnitine | 17 (85%) | 20 (80%) | 17 (77%) | 31 (65%) | 35 (60%) |
| Fructose | **20 (100%)** | **25 (100%)** | **22 (100%)** | **48 (100%)** | **58 (100%)** |
| Fumaric acid | 4 (20%) | 4 (16%) | 1 (5%) | 7 (15%) | 3 (5%) |
| Galactose | 0 (0%) | 0 (0%) | 0 (0%) | 0 (0%) | 1 (2%) |
| Inosine | 0 (0%) | 1 (4%) | 0 (0%) | 1 (2%) | 0 (0%) |
| Isobutyric acid | 14 (70%) | 19 (76%) | 16 (73%) | 26 (54%) | 39 (67%) |
| Ketovaline | **20 (100%)** | **25 (100%)** | **22 (100%)** | **48 (100%)** | **58 (100%)** |
| Pyroglutamic acid | **20 (100%)** | **25 (100%)** | **22 (100%)** | 43 (90%) | 57 (98%) |
| tert-Butanol | **20 (100%)** | **25 (100%)** | 21 (95%) | 37 (77%) | 48 (83%) |

Metabolites that could be detected in all patients of a patient group (100% detection rate) are indicated in bold.

**Figure S1. Venn diagram for metabolites detected in serum (red), CSF (blue) and both matrices.**


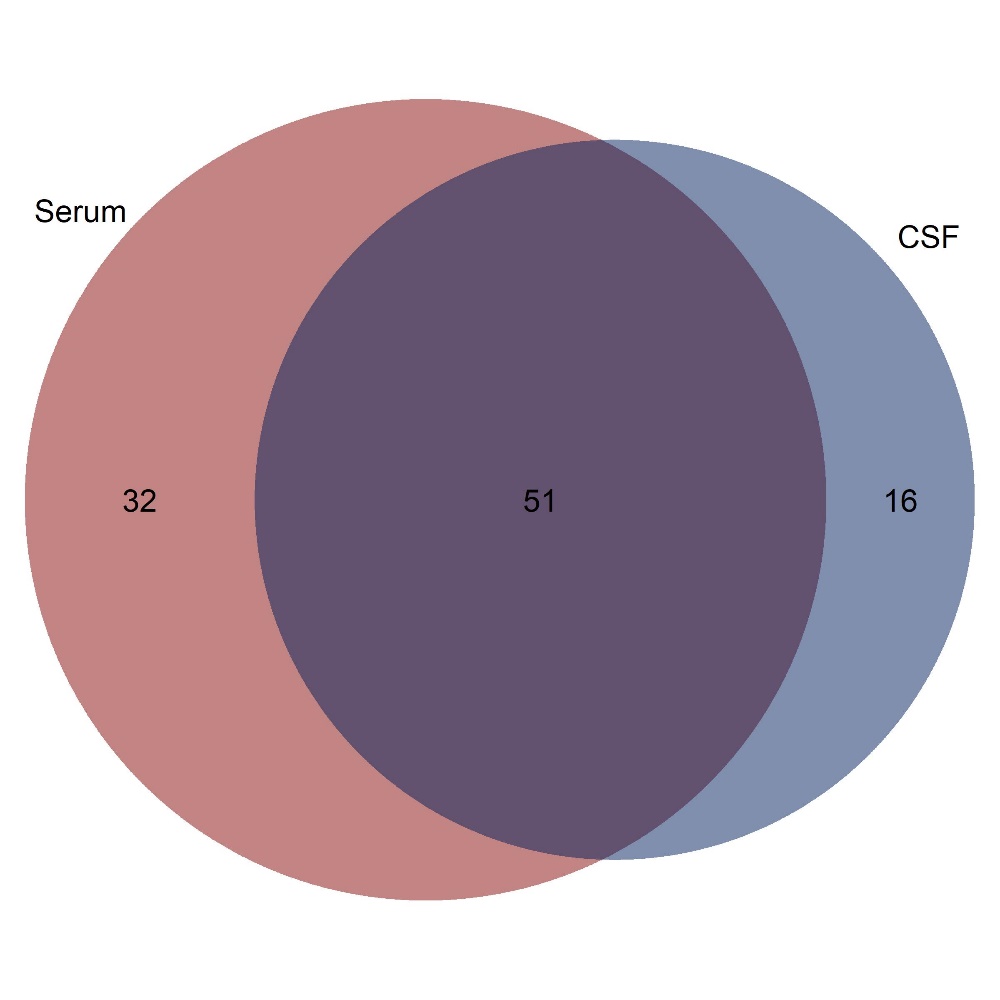


CSF = cerebrospinal fluid.

**Figure S2. Association of metabolite levels in CSF and serum with age in control patients**


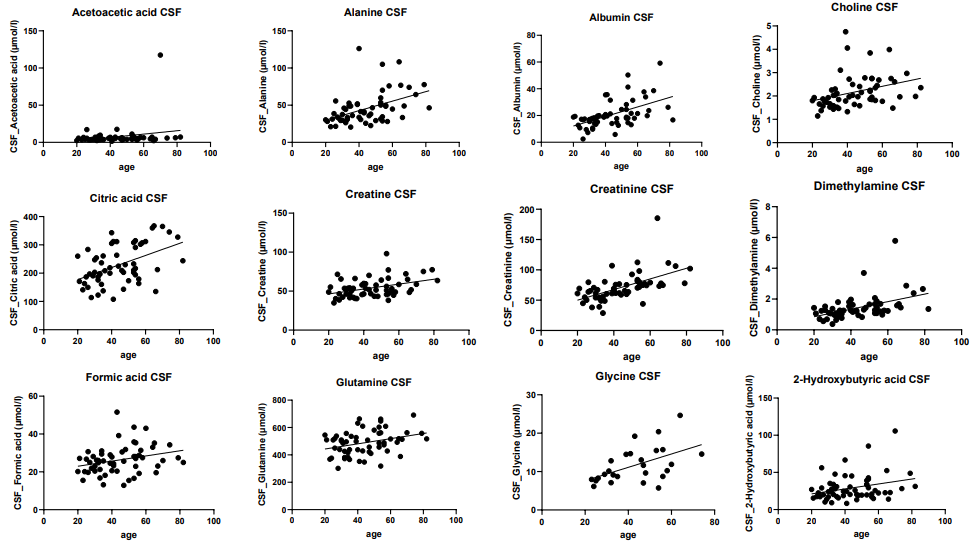


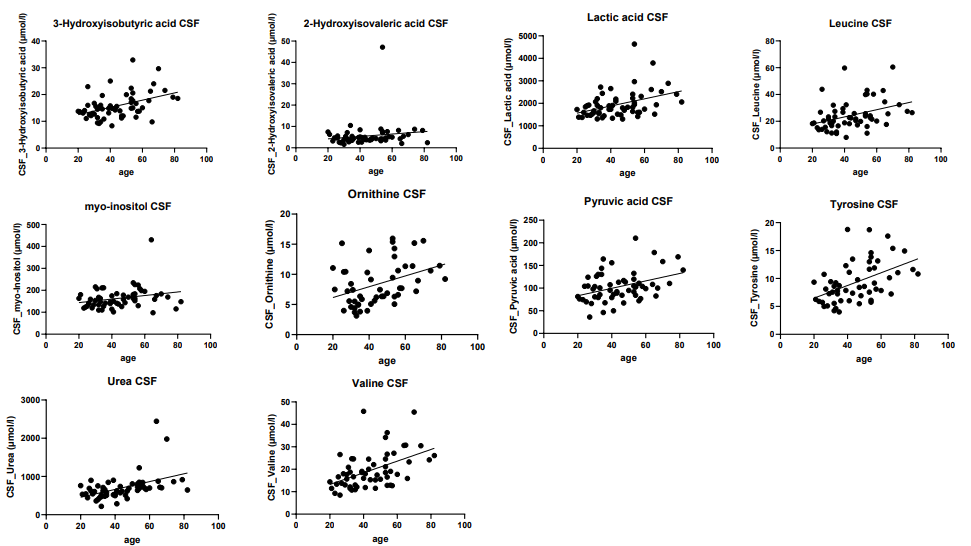


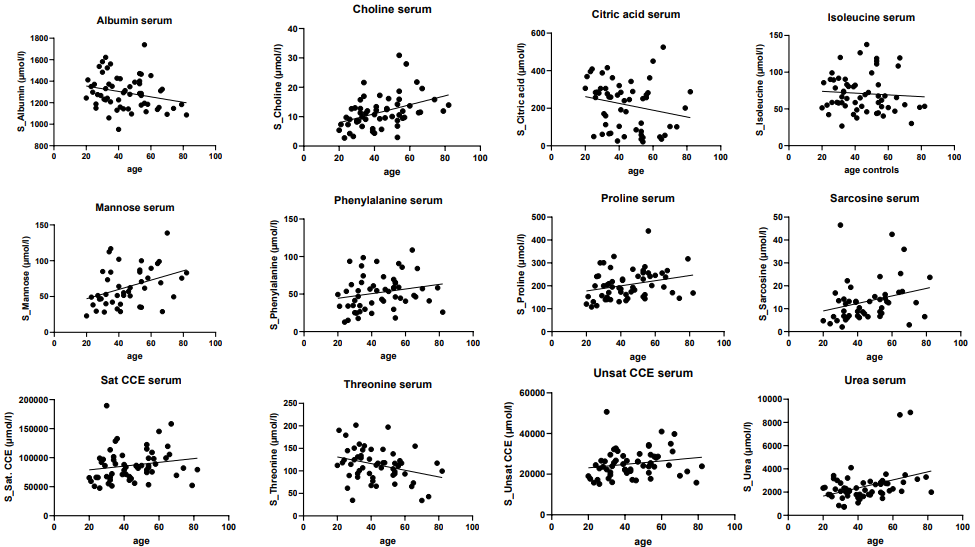


In 58 control patients, associations of 84 metabolites detectable in CSF, serum, or both with age was assessed by plotting the concentrations of metabolites in CSF or serum against age. The figure shows the 34 significant associations of metabolites in CSF and serum with age as assessed by Spearman-Rank analyses. The respective *p* and r values are listed in Supplemental Table 2. The thick line represents the linear regression line.

CSF = cerebrospinal fluid, S = serum.

**Figure S3. Association of CSF/serum metabolite quotients with CSF/serum albumin quotients.**


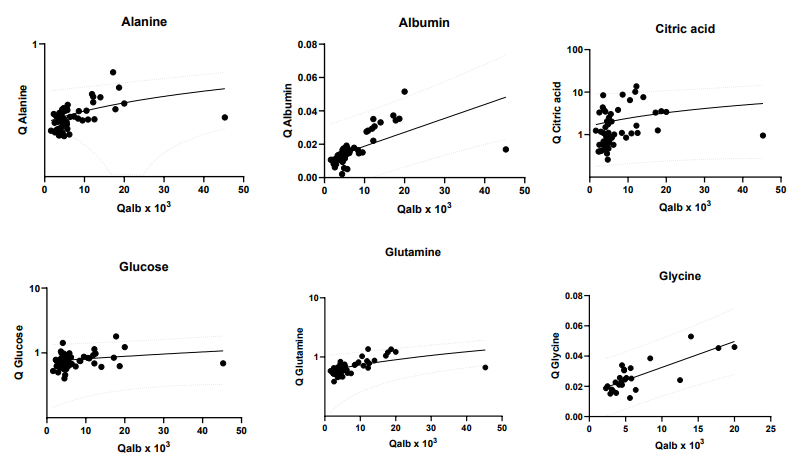


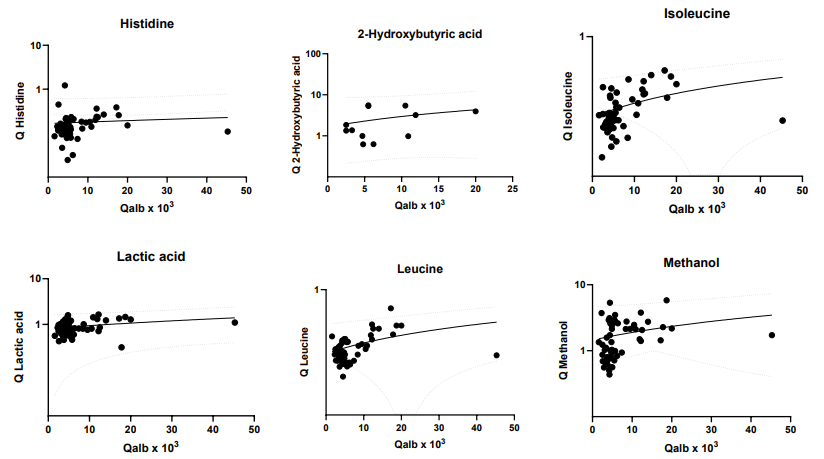


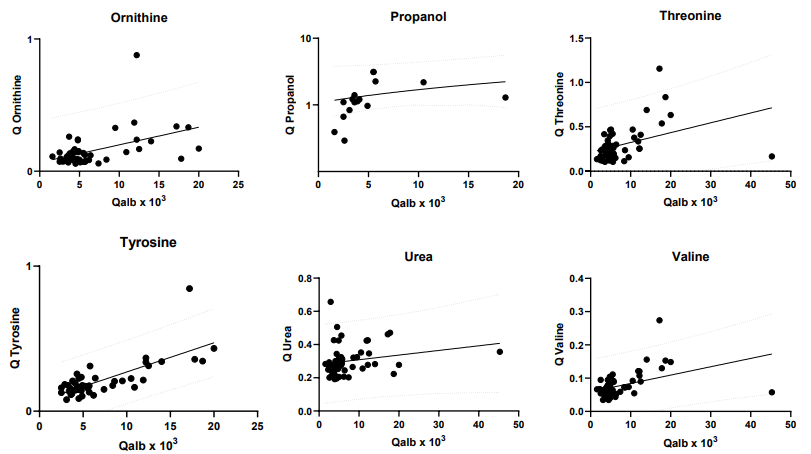


CSF/serum metabolite quotients were plotted against Qalb values of all 48 metabolites detected in both, serum and CSF, of all 58 control patients. The figure shows plots of the 18 CSF/serum metabolites significantly associated with Qalb as assessed by Spearman-rank analysis. The thick line represents the linear regression line, and the dotted lines the upper and lower 99% prediction bands, indicating the area in which 99% of all data points are expected to fall. Please note that the plot for albumin shows the CSF/serum albumin quotient as measured by routine clinical chemistry on the x-axis and as measured by NMR spectroscopy on the y-axis.

Qalb = CSF/serum albumin quotient
